# Controversies in terminology associated with management of BCG‐unresponsive NMIBC in Asia‐Pacific

**DOI:** 10.1111/iju.15298

**Published:** 2023-10-05

**Authors:** Eiji Kikuchi, Chi‐Fai Ng, Hiroshi Kitamura, Ja Hyeon Ku, Lui Shiong Lee, Tzu‐Ping Lin, Junice Yi Siu Ng, Hiroyuki Nishiyama, Darren Ming‐Chun Poon, Ravindran Kanesvaran, Ho Kyung Seo, Carmel Spiteri, Ee Min Tan, Yuh‐Shyan Tsai, Ben Tran

**Affiliations:** ^1^ Department of Urology St. Marianna University School of Medicine Kawasaki Japan; ^2^ Department of Surgery The Chinese University of Hong Kong Hong Kong Hong Kong; ^3^ Department of Urology University of Toyama Toyoma Japan; ^4^ Department of Urology Seoul National University Seoul South Korea; ^5^ Department of Urology Seng Kang General Hospital Singapore Singapore; ^6^ Department of Urology Taipei Veterans General Hospital Taipei Taiwan; ^7^ Health Economics and Outcomes Research IQVIA Asia‐Pacific Singapore Singapore; ^8^ Department of Urology University of Tsukuba Tsukuba Japan; ^9^ Comprehensive Oncology Center Hong Kong Sanatorium & Hospital Hong Kong Hong Kong; ^10^ Division of Medical Oncology National Cancer Centre Singapore Singapore Singapore; ^11^ Department of Urology National Cancer Center Goyang‐si South Korea; ^12^ Market Access Asia Pacific MSD Macquarie Park, NSW Macquarie Park Australia; ^13^ Department of Urology National Cheng Kung University Hospital Tainan Taiwan; ^14^ Department of Medical Oncology Peter MacCallum Cancer Centre Melbourne Australia

**Keywords:** BCG vaccine, neoplasm recurrence, patient care management, practice guidelines as topic, urinary bladder neoplasms

## Abstract

**Objectives:**

Examine the understanding of terminologies and management patterns of bacillus Calmette‐Guérin (BCG)‐unresponsive nonmuscle invasive bladder cancer (NMIBC) in six territories in Asia‐Pacific.

**Methods:**

This study involved two phases: (1) a survey with 32 urologists and 7 medical oncologists (MOs) and (2) a factorial experiment and in‐depth interviews with 23 urologists and 2 MOs. All clinicians had ≥8 years' experience managing NMIBC patients in Australia, Hong Kong, Japan, South Korea, Singapore, and Taiwan. Data from Phase 1 were summarized using descriptive statistics; content and thematic analyses applied in Phase 2.

**Results:**

In phase 1, 35% of clinicians defined BCG‐unresponsive as BCG‐refractory, ‐relapse and ‐resistant, 6% defined it as BCG‐refractory and ‐relapse; 22% classified BCG‐failure as BCG‐refractory, ‐relapse, ‐resistant, and when muscle‐invasive bladder cancer is detected. If eligible and willing, 50% (interquartile range [IQR], 50%–80%) of BCG‐unresponsive patients would undergo radical cystectomy (RC), and 50% (IQR 20%–50%) of RC‐eligible patients would receive bladder‐sparing treatment or surveillance. In phase 2, we found that 32%, 88%, and 48% of clinicians, respectively, used “BCG‐unresponsive,” “BCG‐refractory,” and “BCG‐relapse” in clinical practice but with no consistent interpretation of the terms. Compared with EAU definitions, 8%–60% of clinicians appropriately classified 9 tumor types that are persistent or recurrent after adequate BCG. Fifty percent of clinicians mentioned a lack of bladder‐preserving treatment that outperforms RC in quality of life as a reason to retreat BCG‐unresponsive patients with BCG.

**Conclusions:**

Our study revealed varied understanding and application of BCG‐unresponsive terminologies in practice. There is a need for a uniform and simple definition of BCG‐unresponsive disease in Asia‐Pacific.

**Abbreviations & Acronyms**
BCG: bacillus Calmette‐Guérin
CIS: carcinoma in situ
EAU: European Association of Urology
FDA: Food and Drug Administration
HG: high‐grade
IBCG: International Bladder Cancer Group
IQR: interquartile range
IVC: intravesical chemotherapy
JUA: Japanese Urological Association
NMIBC: nonmuscle‐invasive bladder cancer
RC: radical cystectomy

## INTRODUCTION

Bladder cancer is prevalent in Asia‐Pacific, with 212 014 new cases diagnosed in 2020.^1^ Approximately 75% of newly diagnosed bladder cancer patients have nonmuscle‐invasive bladder cancer (NMIBC).^2^ The current standard of care is transurethral resection of bladder tumor, followed by intravesical Bacille Calmette–Guérin (BCG) and intravesical chemotherapy (IVC). High‐risk NMIBC is characterized by frequent recurrence and a high risk of progression despite adequate treatment.^3^

Clinical practice guidelines (Data S1) recommend the use of various terms to describe patients who fail or do not respond to BCG, such as BCG failure and BCG‐unresponsive. Differences exist among guidelines on the classification of BCG failure and definitions of BCG‐unresponsive. The Food and Drug Administration (FDA),^4^ but not the European Association of Urology (EAU),^5^ explicitly deems T1 high‐grade disease at first evaluation after an induction course of BCG as BCG‐unresponsive. EAU, International Bladder Cancer Group (IBCG) and Japanese Urological Association (JUA), defines BCG relapse as recurrence of high‐grade tumors after completion of adequate therapy, despite an initial response.^5^, ^6^, ^7^ However, IBCG and JUA further differentiated this term into early, intermediate, and late relapse.^6^, ^7^ Variability in the definitions available may result in a diverse understanding of these terms and disease management. A consequence of differing understanding of terminology is a variability in patient care, possibly causing suboptimal treatment.

There is a paucity of research examining the understanding of terminologies and clinical management related to BCG‐unresponsive NMIBC, especially in Asia‐Pacific. This work sought to examine physicians' understanding of definitions, such as those outlined in the EAU guidelines, related to patients who fail or do not respond to BCG and corresponding management approaches.

## METHODS

This study involved a 2‐phase mixed‐method sequential explanatory approach. An initial quantitative survey was used to collect data on characteristics deemed BCG‐unresponsive and management options for BCG‐unresponsive patients. We hypothesized that clinicians could be providing appropriate treatment but might not be conversant with terminologies related to BCG‐unresponsiveness and failure. A qualitative approach was used in phase 2 to seek further explanation of phase 1 results through a factorial experiment and semistructured interview.

### Phase 1: Quantitative survey

Clinicians from Australia, Hong Kong, Japan, South Korea, Singapore, and Taiwan with at least 8 years' experience managing patients with NMIBC and spending at least 50% of their time in direct patient care were deemed eligible. Urologists and medical oncologists were identified from a commercial panel of clinicians who had previously participated in similar surveys and were invited to participate in this survey.

A self‐administered survey was developed through a literature review and discussion with 12 domain experts (co‐authors of this paper). Respondents were given predetermined options and a free‐text option. The questionnaire (Data S2) consisted of two main sections: (1) clinician understanding of BCG‐unresponsive and BCG failure; and (2) how respondents managed BCG‐unresponsive patients. Respondents could opt out of question(s) not within their expertise. The questionnaire was translated into local languages and piloted with two eligible clinicians then implemented via an online survey platform.

### Phase 2: Factorial experiment

A factorial experiment, via semistructured interview, was conducted with a subset of respondents who completed phase 1. To test their clinical decision‐making process, vignettes were developed that consisted of nine clinical scenarios of tumor characteristics varying in size, grade, and time of recurrence after adequate BCG (Data S3). After viewing each vignette, respondents were asked how they would manage the case and if they would consider the case BCG‐unresponsive or failure. They were then asked how they would manage a case deemed “BCG‐unresponsive” and what factors they considered.

Moderators were trained in the experimenting process and had no knowledge of the respondent's results from phase 1. The experiments were conducted via online video conference and in participants' native language. Interviews were conducted within 2 months after Phase 1 survey to avoid recall bias.

#### Data analysis

Quantitative data from the phase 1 survey were summarized using descriptive statistics. Data from the phase 2 factorial experiment were transcribed and translated into English. The data were analyzed using content and thematic analyses. Thematic analysis provides an interpretation of participants' meanings, while content analysis is a direct representation of participants' responses and the number of occurrences of content (keywords) in the data. Responses on management approaches and understanding of terminologies were compared with the EAU guidelines (Data S1), as clinicians in the region were likely to be familiar with the EAU guidelines.

## RESULTS

A total of 39 respondents participated in phase 1. Among them, 25 took part in phase 2 (Table 1). Majority of participants were practicing urologists.

**TABLE 1:** Characteristics of survey (*N* = 39) and factorial experiment participants (*N* = 25).

Characteristics	Phase 1 survey^a^ (*N* = 39)	Phase 2 factorial experiment (*N* = 25)
	*n* (%)	*n* (%)
Country/region		
Australia	8 (21)	5 (20)
Hong Kong	3 (8)	3 (12)
Japan	9 (23)	6 (24)
Korea	8 (21)	5 (20)
Taiwan	6 (13)	3 (12)
Singapore	5 (15)	3 (12)
Specialty^b^		
Urologist	32 (82)	23 (92)
Medical oncologist	7 (18)	2 (8)
Practice		
Public only	17 (44)	10 (40)
Private only	20 (51)	13 (52)
Both public and private	2 (5)	2 (8)

^a^ In the survey, responses were obtained from all urologists while medical oncologists were given the option to opt‐out of questions that were not within their experience or practice.

^b^ In the factorial experiment, medical oncologists were from Hong Kong (*n* = 1) and Australia (*n* = 1).

### How do physicians classify BCG‐unresponsive and its related terms?

#### Survey findings

**FIGURE 1:**
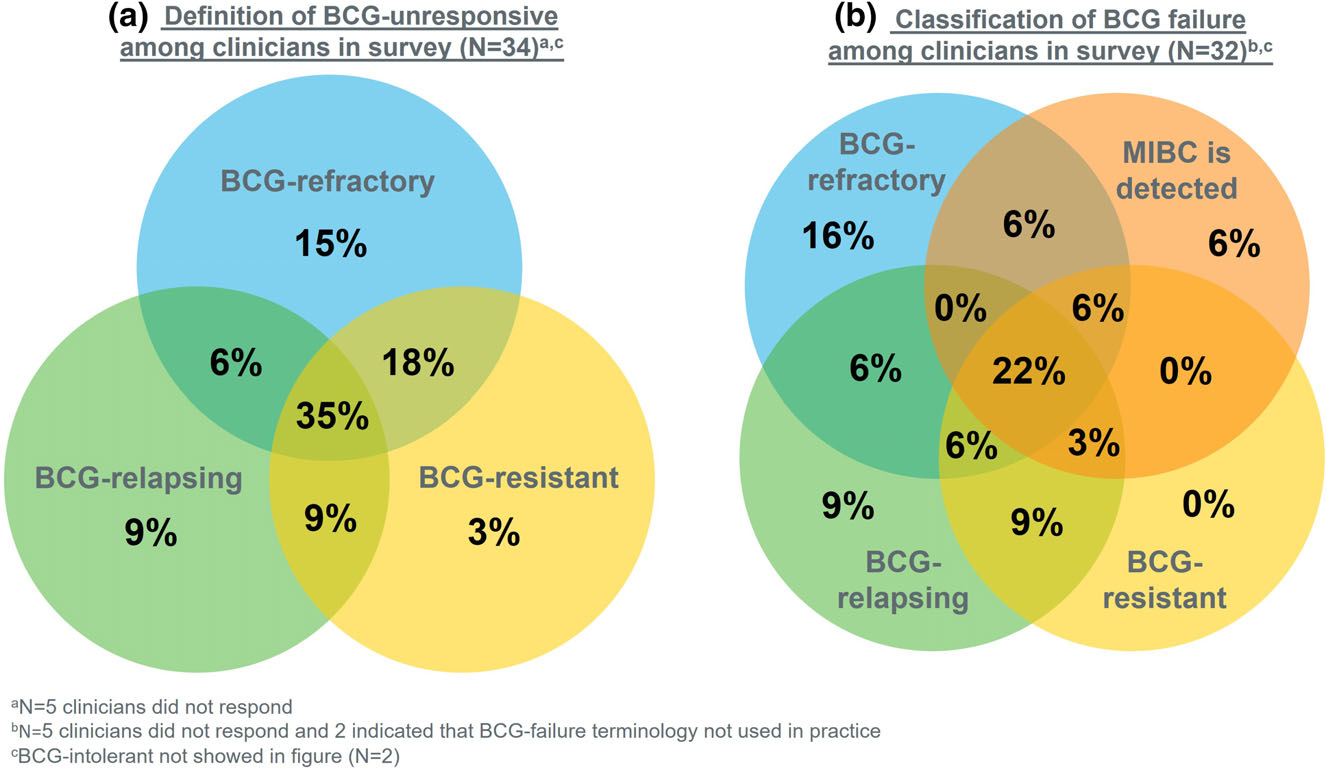
Definition of (a) BCG‐unresponsive (*N* = 34) and (b) classification of BCG failure (*N* = 32) among clinicians in the survey. BCG, bacillus Calmette–Guérin; MIBC, muscle‐invasive bladder cancer.

Figure 1 shows how respondents classified “BCG‐unresponsive” and “BCG failure” in their practice. 6% (*n* = 2/34) defined “BCG‐unresponsive” as BCG‐refractory and ‐relapsing, while 35% (*n* = 12/34) of respondents defined it as BCG‐refractory, BCG‐relapsing, and BCG‐resistant, 18% (*n* = 6/34) defined it as BCG‐refractory and BCG‐resistant, 15% (*n* = 5/34) defined it as BCG‐refractory only, 9% (*n* = 3/34) defined it as BCG‐relapsing only, and 9% (*n* = 3/34) defined it as BCG‐relapsing and BCG‐resistant. Of the 34 respondents, two indicated that they did not use the “BCG failure” in their practice. Of remaining respondents, 22% (*n* = 7/32) classified “BCG failure” as BCG‐refractory, BCG‐relapsing, BCG‐resistant, and whenever muscle‐invasive bladder cancer is detected. Only two respondents considered BCG‐intolerant as BCG‐unresponsive and BCG failure (not shown in Figure 1). Clinicians' understanding of the definition of BCG‐resistant is shown in Figure S1.

#### Qualitative findings

**FIGURE 2:**
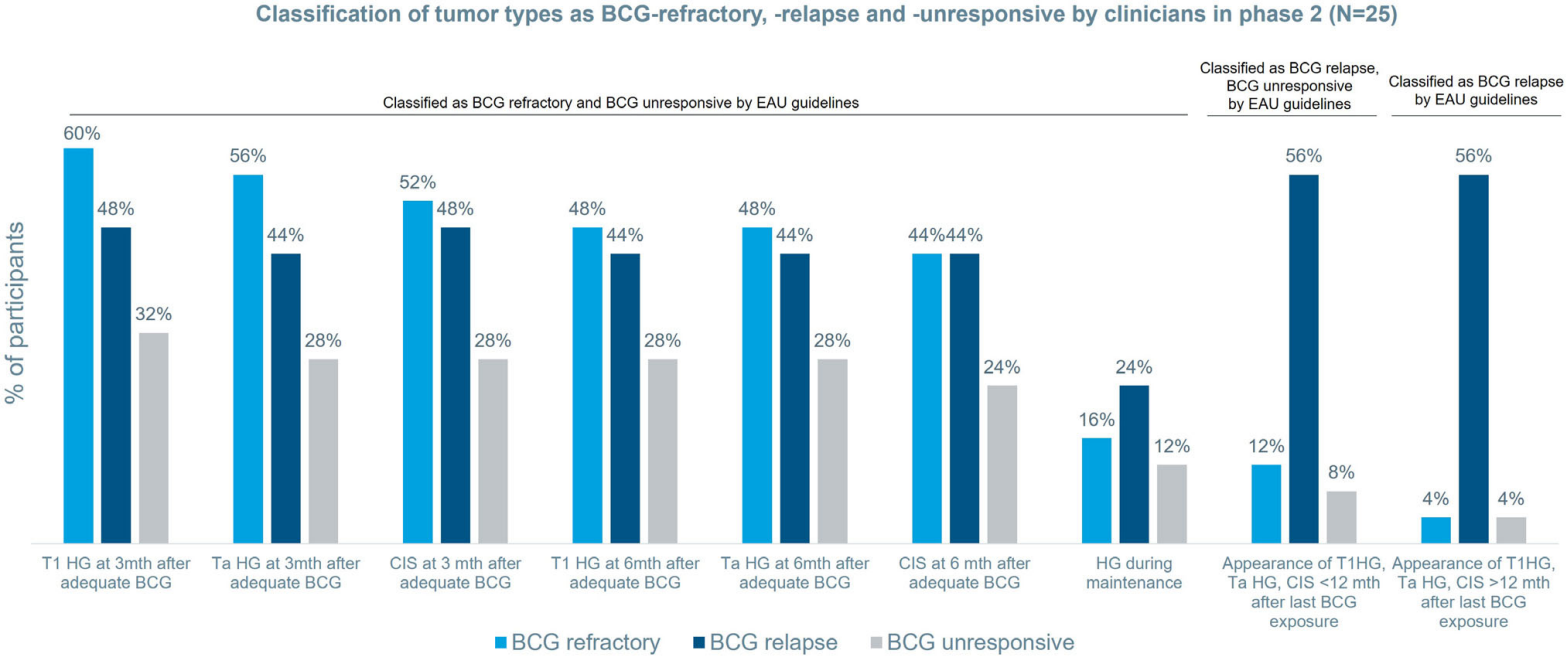
Classification of tumor types as BCG‐refractory, ‐relapse, and ‐unresponsive by clinicians in phase 2 (*N* = 25). BCG, bacillus Calmette–Guérin; CIS, carcinoma in situ; HG, high‐grade; mth, month. Participants could select more than one option in the survey.

Given the varied responses to definitions of BCG‐unresponsiveness and ‐failure in phase 1, clinicians' understanding of BCG‐refractory, −relapsing, and −resistant were explored in phase 2. Figure 2 shows clinicians' classification of noninvasive (Ta) high‐grade (HG) papillary carcinoma, HG carcinoma that has invaded into the lamina propria (T1), carcinoma in situ (CIS) tumors that recur at 3 or 6 months after adequate BCG or the appearance of tumors within or beyond 12 months after last BCG exposure as BCG‐refractory, BCG‐relapse, and BCG‐unresponsive. BCG‐unresponsive was the terminology least used to classify all tumor types. Compared with EAU guidelines, up to 60% of clinicians appropriately classified tumor characteristics as BCG‐refractory, while 40% of clinicians also classified these tumors as BCG‐relapse. 24% of clinicians classified the appearance of an HG tumor during BCG maintenance as BCG‐relapse, and 16% classified it as BCG‐refractory. 56% of clinicians appropriately classified appearance of Ta, T1, and CIS tumors within and beyond 12 months of last BCG exposure as BCG‐relapse (Figure 2).

We found in phase 2 that one‐third of participants applied “BCG‐unresponsive” (32%, *n* = 8/25), “BCG failure” (36%, *n* = 9/25), while 88% (*n* = 22/25) applied “BCG‐refractory” in practice. Those who used “BCG‐unresponsive” in practice equated it to “BCG‐refractory” (*n* = 9/25). Some (*n* = 4/25) thought it equated to “BCG‐relapse,” “development of CIS” (within 12 months of adequate BCG exposure) (*n* = 2/25), and “worsening of disease” (*n* = 2/25). One spelled out that “BCG‐unresponsive disease is defined by several criteria approved by the FDA.”

Nine participants (*n* = 9/25) pointed out that BCG failure is an umbrella term to describe when disease cannot be controlled with BCG, including intolerance. Some (*n* = 4/25) acknowledged they were unable to distinguish BCG failure from BCG‐relapse, BCG‐refractory, and BCG‐unresponsive. Upon further probing, 80% (*n* = 20/25) mentioned that “BCG‐unresponsive and BCG failure” are interchangeable.

**Table:** 

*BCG failure is a broad term that encompasses BCG‐unresponsive and other terms*. *~ Urologist 2, Australia* *I mean, how could I explain that “BCG‐unresponsive” is different from “BCG‐refractory” or “BCG‐failure”? They're all similar terms*. *~ Urologist 5, South Korea* *I think it just means exactly the same thing (between unresponsive and failure). Depending on the clinical situation, you can pretty much use in the same way*. *~ Medical Oncologist 3, Australia*

Two participants elaborated the differences.

**Table:** 

*Any time there's either recurrence or progression after you've had time where it was clear, then that's failure. If it never got better and you kept on getting recurrence and you never had a period where there was clear cystoscopy, then it's unresponsive*. *~ Urologist 6, Australia* *It's called ‘BCG‐unresponsive’ when the patient's body condition has never been fine on endoscopy despite the use of BCG. However, if it's okay for a while and then recurs after 3, 6, or 9 months, you can think of it as a BCG failure*. *~ Urologist 2, South Korea*

Twenty‐seven percent (*n* = 6/22) of participants who used “BCG‐refractory” in their practice defined it as when the tumor does not respond to BCG within 3 to 6 months, and 23% (*n* = 5/22) defined it as recurrence of the tumor within 3 to 12 months after BCG treatment.

Twenty percent (*n* = 5/25) of participants were not able to differentiate refractory from relapse, expressing that the terms had little implications on clinical practice. Forty percent (*n* = 10/25) of participants distinguished between early and late relapse, defining early relapse as a recurrence within 12 months of last BCG administration. Two participants used the term “relapse” in practice but did not distinguish between early or late.

**Table:** 

*As for early relapse and late relapse… I don't think I have divided them like that much. But it's said that if the condition relapsed at the 1st cycle after BCG, it's known that the risk of progression is higher. So, I can distinguish them like that*. *~ Urologist 1, South Korea* *I think it is difficult to distinguish between refractory and relapse*. *~ Urologist 3, Taiwan* *(My) impression that it (relapse) is similar to refractory. Early is considered within three months. I do not use that term because it is very close in meaning*. *~ Urologist 1, Japan*

### How do clinicians manage tumor persistence or recurrence after BCG?

#### Survey findings

Respondents indicated that 50% (interquartile range [IQR] 50%–80%) of their BCG‐unresponsive patients received RC if they were eligible and willing to undergo RC. 50% (IQR 20–50%) of BCG‐unresponsive patients who are eligible for RC would receive bladder‐sparing treatment options or undergo further surveillance (Table S1). For BCG‐unresponsive patients who are eligible but unwilling to undergo RC (Figure S2), respondents most frequently gave BCG re‐treatment (34%), and IVC (26%) immediately, post BCG unresponsive. Twenty‐three percent of respondents would not provide further treatment. 80% of respondents would choose RC after first line of pharmacological treatment in BCG‐unresponsive patients.

#### Qualitative findings

**FIGURE 3:**
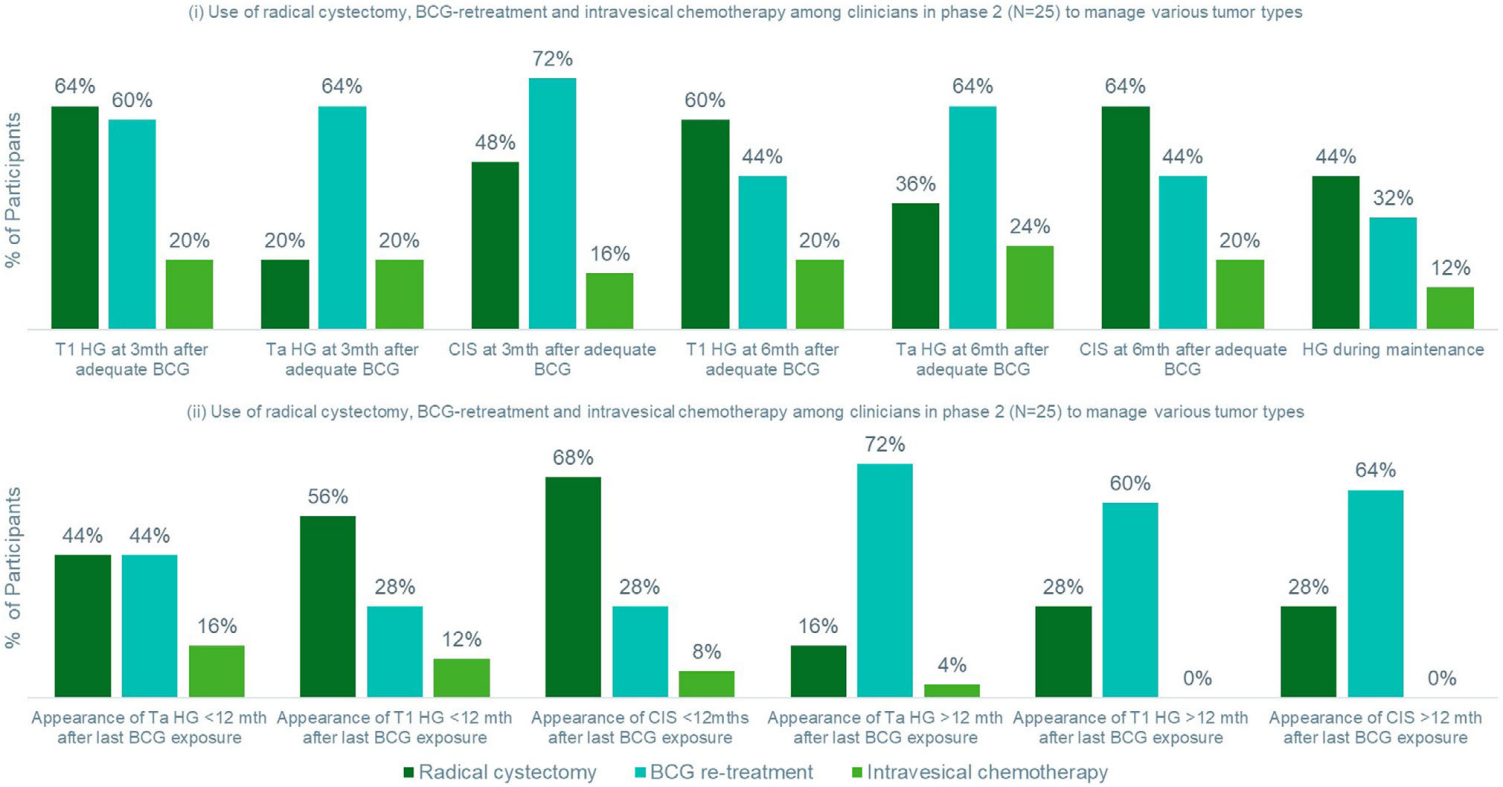
Use of radical cystectomy, BCG‐retreatment, and intravesical chemotherapy among clinicians in phase 2 (*N* = 25) to manage various tumor types. BCG, bacillus Calmette–Guérin; CIS, carcinoma in situ; HG, high‐grade; mth, month. Participants could select more than one option.

Given the wide variation in clinicians' understanding of “BCG‐unresponsive”, we sought to explore clinical decisions based on different BCG‐unresponsive tumor characteristics using a factorial experiment in phase 2. Figure 3 shows the proportion of participants who would respectively manage the tumors with RC, BCG re‐treatment, and IVC. BCG‐retreatment was preferred for TaHG, CIS tumors that recurred at 3 months, and TaHG tumors that recurred at 6 months after adequate BCG exposure. RC was preferred for T1HG that recurred at 3 or 6 months, CIS tumors that recurred 6 months after adequate BCG exposure, and HG tumors that appear during maintenance. More participants would manage the appearance of T1HG and CIS tumors within 12 months of last BCG exposure with RC than with BCG re‐treatment. A similar proportion of participants would use RC and BCG re‐treatment for TaHG tumors that appear within 12 months of the last BCG exposure. Participants preferred BCG re‐treatment over RC for TaHG, T1HG, and CIS after 12 months of the last BCG exposure.

We found that those who supported BCG re‐treatment justified it as a standard of care and that current evidence supports the use of BCG (*n* = 10/25, 40%). When probed why BCG re‐treatment is still provided among BCG‐unresponsive patients, many cited a lack of effective alternative bladder‐preserving treatment options (*n* = 13/25, 52%), the low cost of BCG compared to other treatments (*n* = 3/25, 12%), and outperforming RC in terms of quality of life, giving hope for those who refused RC (*n* = 7/25, 28%).

**Table:** 

*I would do it (BCG re‐treatment) since there is no other option other than BCG. Anyway, it is scientifically proven to be effective. Though it cannot get rid of the tumor, BCG could delay the tumor from recurring or growing*. *~ Urologist 3, South Korea* *You are committing them to a therapy, which they've already had, so it's an easier sell. You've selected a population that is already compliant with BCG, so you're giving them something they already know and oftentimes these are patients who want to keep their bladders or aren't fit to have a cystectomy. It's a sample population that's slightly skewed toward re‐induction*. *~ Urologist 2, Australia*

Most common reasons for use of IVC in BCG‐unresponsive patients include nonresponse to BCG despite a few induction courses (*n* = 9/14), intolerance to BCG (*n* = 4/14), shortage of BCG (*n* = 2/14), patient preference (*n* = 2/14), or perceived efficacy of IVC for tumors without very high‐risk features (*n* = 1/14).

**Table:** 

*BCG comes first for patients with CIS. I think the rest of patients should receive IVC*. *~ Urologist 4, South Korea* *Chemotherapy is widely given to patients who want it*. *~ Urologist 2, Japan* *If there's signs of early BCG failure, then we're more likely to aim for IVC over re‐induction*. *~ Urologist 2, Australia*

## DISCUSSION

Our study revealed wide variations in clinicians' understanding of terminologies related to BCG treatment, with discrepancies between their understanding and that stated in guidelines. Some clinicians did not apply these terminologies, as they felt there was limited practical implication of these terms on patient management. Management of patients with BCG‐unresponsive tumors also varied with tumor type, timing of recurrence from the last BCG exposure, and patient willingness to undergo RC.

Clinical practice guidelines aim to standardize and optimize patient care and put forward recommendations informed by a systematic review of evidence. Terminologies used in guidelines are thus crucial to defining applicable patient populations and guiding management approaches.

BCG‐unresponsive is a concept developed to identify patients unlikely to respond to further BCG therapy and to homogenize the inclusion criteria for clinical trials.^8^ However, with the heterogeneous nature of the disease, variations exist in how BCG‐unresponsive is defined by the FDA and various guidelines.^9^, ^10^, ^11^, ^12^ According to EAU guidelines, BCG‐unresponsive NMIBC includes BCG‐refractory and early‐relapsing tumors.^5^ The inclusion of a diverse population has rendered this terminology nondiscriminative and difficult to apply in research and clinical practice.^13^ Variations in clinicians' understanding of BCG‐unresponsive observed in our study could be due to the application of different guidelines in the regions and practice differences such as intervals of patient assessment after BCG administration and differing access to BCG due to cost or BCG shortage. At the time of this survey, no treatment was approved for BCG‐unresponsive high‐risk NMIBC in Asia‐Pacific, and the concept was consequently less meaningful and applicable to clinicians. Ongoing phase 3 trials evaluating the safety and efficacy of immuno‐oncology drugs for BCG‐unresponsive NMIBC may hold promising alternatives for these patients.^14^ Treatment patterns for BCG‐unresponsive NMIBC may further vary across the region depending on the approval and reimbursement of novel treatment options.^4^

Controversies in terminologies and the resultant lack of understanding could have led to variability in the treatment approaches identified in our study, implying suboptimal treatment and an even greater burden on patients. Notably, although guidelines recommend these patients be managed with RC and not additional BCG,^10^ RC and BCG re‐treatment were approaches commonly mentioned by participants. In practice, RC is often refused by patients due to the risk of complications and poor quality of life.^15^, ^16^, ^17^ There remains mixed evidence on the efficacy of BCG rechallenge and IVC for BCG‐unresponsive disease, further complicated by the heterogeneity in clinical trial designs.^18^ Consistent with recent literature on real‐world treatment patterns,^19^, ^20^ our study found that aside from IVC, BCG rechallenge, radiation therapy, and surveillance were main alternative approaches for BCG‐unresponsive patients. This could be attributed to limited bladder‐sparing options that are effective and tolerable, highlighting an unmet need for effective treatments that delay disease progression. Ongoing trials on novel bladder‐sparing agents and delivery mechanisms would be timely for BCG‐unresponsive patients who are ineligible or unwilling to undergo RC.^21^

This study should be interpreted within the following limitations. The study was based on physicians' experiences and preferences and was not designed to consider individual patient characteristics. We were unable to validate clinicians' responses with their treatment patterns. These findings are exploratory, given the small number of clinicians surveyed, and are not intended to guide treatment decisions. Nonetheless, to our knowledge, this is the first study assessing clinicians' level of understanding of terminologies related to BCG‐unresponsive NMIBC and one of the few comparing treatment approaches for BCG‐unresponsive diseases against an established clinical practice guideline.^22^ While it is challenging to capture all clinical scenarios in a questionnaire, a qualitative component allowed us to comprehend the nuances of clinicians' understanding in this therapeutic area.

Our study revealed a varied understanding of BCG‐unresponsive terminologies in practice, highlighting the difficulty in applying definitions and recommendations in existing guidelines. The need for a uniform and concise definition of BCG‐unresponsive disease in the APAC region increases as novel therapies emerge. Efforts to standardize BCG‐unresponsive disease identification and management could pave the way for more interventions that improve patient outcomes and quality of life.

## AUTHOR CONTRIBUTIONS

All authors were involved in the conception and design of this study, interpretation of the results, and critical revision of the manuscript. Ng Junice Yi Siu and Tan Ee Min were involved in data analysis and drafting the manuscript, with direction from Kikuchi Eiji, Ng Chi‐Fai and Tran Ben who supervised the study. All authors read and approved the final manuscript.

## CONFLICT OF INTEREST STATEMENT

LLS received research funding from Janssen, honoraria from AstraZeneca, MSD, Bayer, Astellas, and Janssen; played an advisory role for AstraZeneca, MSD, Bayer, Astellas, and Janssen. KE received consulting fees and honoraria from MSD. LTP received honoraria from Astellas, MSD, Ferring pharmaceuticals, Janssen, Bayer, and AstraZeneca; received Docetaxel/Cabazitaxel compounds from Sanofi. KH received consulting fees from Kissei and Takeda; received honoraria from Astellas, AstraZeneca, MSD, Sanofi, Takeda, Bayer, Bristol Myers Squibb, Merck Biopharma, and Janssen; played an advisory role for Janssen, Astellas, MSD, and Pfizer. NCF received consulting fees from Cornerstone Medical and Agilis; received honoraria from Boston Scientific, Janssen, Olympus, Bayer, and Ipsen; played an advisory role for Newlife Medical Limited, Jiangsu Hengrui Medicine and Nonagen. He also received equipment materials/drugs from Olympus and Janssen. KR received consulting fees from MSD, AstraZeneca, Bristol‐Myers Squibb, Eisai, Astellas, Johnson & Johnson, and Pfizer; received honoraria from MSD, AstraZeneca, Bristol‐Myers Squibb, Astellas, Pfizer, and Johnson & Johnson. TB received research fundings Bristol‐Myers Squibb, MSD, Ipsen, Astellas Pharma, Janssen‐Cilag, Amgen, Pfizer, Genentech, AstraZeneca, and Bayer. He received honoraria from Astellas Pharma, MSD, Janssen‐Cilag, Sanofi, Tolmar, Amgen, Bristol‐Myers Squibb, Pfizer, Janssen, and Bayer; received travel grants from Amgen and Astellas Pharma; played an advisory role for Amgen, Astellas Pharma, Bayer, Sanofi, Tolmar, Janssen‐Cilag, Bristol‐Meyers Squibb, Ipsen, MSD Oncology, IQVIA, Novartis, Pfizer/EMD Serono, AstraZeneca, and Roche Molecular Diagnostic. NH received research grants from Astellas, MSD, Bayer, Ono Pharmaceutical and Takeda Pharmaceutical; received honoraria from MSD, Chugai Pharmaceutical and Astellas. NJYS and TEM are full‐time employees of IQVIA, that was commissioned to carry out this study. SC was a full‐time employees of MSD. KJH, PDMC, SHK, and TYS received honoraria from MSD and have no other conflicts of interest to declare. This work was supported by funding from MSD International GmbH (Singapore). The funding source had no role in the analysis of this study.

## ANIMAL STUDIES

N/A.

## APPROVAL OF RESEARCH PROTOCOL AND INFORMED CONSENT

This study protocol was reviewed and approved by the following research ethics committees: Melbourne Health Human Research Ethics Committee (HREC/75511/MH‐2021), Hong Kong Society of Uro‐Oncology Medical Council, St Marianna's University Ethics Board, Seoul National University Hospital IRB (H‐2104‐053‐1210), SingHealth Centralized Institutional Review Board (IRB) (2021/2124), and Taipei Veterans General Hospital IRB (2021–06‐002 BC). Informed consent was obtained from all respondents.

## REGISTRY AND REGISTRATION NO. OF STUDY/ TRIAL

N/A.

## Supporting information


Figure S1.

Figure S2.

Table S1.

Data S1.

Data S2.

Data S3.

## References

[1] WHO . Cancer fact sheets: bladder cancer. Lyon. France: International Agency for Research on Cancer, World Health Organization; 2020.

[2] Cavaliere C , D'Aniello C , Cecere S , Di Napoli M , Berretta M , Franco R . Non muscle invasive bladder cancer treatment. World Cancer Res J. 2014;1(1):e126.

[3] Veeratterapillay R , Heer R , Johnson MI , Persad R , Bach C . High‐risk non‐muscle‐invasive bladder cancer—therapy options during intravesical BCG shortage. Curr Urol Rep. 2016;17:68. 27492610 10.1007/s11934-016-0625-z PMC4980405

[4] US Food and Drug Administration . BCG‐unresponsive nonmuscle invasive bladder cancer: developing drugs and biologics for treatment guidance for industry [Internet]. Center for Drug Evaluation and Research 2018. Available from: https://www.fda.gov/media/101468/download#:~:text=Patients%20with%20BCG%2Dunresponsive%20NMIBC,with%20CIS%2C%20or%20CIS%20alone

[5] Babjuk M , Burger M , Capoun O , Cohen D , Compérat EM , Dominguez Escrig JL , et al. European Association of Urology guidelines on non‐muscle‐invasive bladder cancer (Ta, T1, and carcinoma in situ). Eur Urol. 2022;81(1):75–94. 34511303 10.1016/j.eururo.2021.08.010

[6] Matsumoto H , Shiraishi K , Azuma H , Inoue K , Uemura H , Eto M , et al. Clinical practice guidelines for bladder cancer 2019 edition by the Japanese Urological Association: revision working position paper. Int J Urol. 2020;27(5):362–368. 32172529 10.1111/iju.14210

[7] Kamat AM , Colombel M , Sundi D , Lamm D , Boehle A , Brausi M , et al. BCG‐unresponsive non‐muscle‐invasive bladder cancer: recommendations from the IBCG. Nat Rev Urol. 2017;14(4):244–255. 28248951 10.1038/nrurol.2017.16

[8] Defining BCG‐Unresponsive and The Pathway to Clinical Trials in Treating NMIBC ‐ Ashish Kamat [Internet]. [cited 2022 Aug 20]. Available from: https://www.urotoday.com/video‐lectures/bladder‐cancer/video/1179‐interview‐petros‐grivas‐and‐ashish‐kamat.html

[9] Roumiguié M , Kamat AM , Bivalacqua TJ , Lerner SP , Kassouf W , Böhle A , et al. International bladder cancer group consensus statement on clinical trial design for patients with bacillus Calmette‐Guérin‐exposed high‐risk non‐muscle‐invasive bladder cancer. Eur Urol. 2022 Jul;82(1):34–46. 34955291 10.1016/j.eururo.2021.12.005

[10] Shore ND , Palou Redorta J , Robert G , Hutson TE , Cesari R , Hariharan S , et al. Non‐muscle‐invasive bladder cancer: an overview of potential new treatment options. Urol Oncol Semin Orig Investig. 2021;39(10):642–663. 10.1016/j.urolonc.2021.05.015 34167873

[11] Chang SS , Boorjian SA , Chou R , Clark PE , Daneshmand S , Konety BR , et al. Diagnosis and treatment of non‐muscle invasive bladder cancer: AUA/SUO guideline. J Urol. 2016;196(4):1021–1029. 27317986 10.1016/j.juro.2016.06.049

[12] Power NE , Izawa J . Comparison of guidelines on non‐muscle invasive bladder cancer (EAU, CUA, AUA, NCCN, NICE). Bladder Cancer. 2016;2(1):27–36. 27376122 10.3233/BLC-150034 PMC4927900

[13] Shirakawa H , Kikuchi E , Tanaka N , Matsumoto K , Miyajima A , Nakamura S , et al. Prognostic significance of bacillus Calmette‐Guérin failure classification in non‐muscle‐invasive bladder cancer. BJU Int. 2012;110(6b):E216–E221. 22313616 10.1111/j.1464-410X.2011.10894.x

[14] Kim HS , Seo HK . Emerging treatments for bacillus Calmette–Guérin‐unresponsive non‐muscle‐invasive bladder cancer. Investig Clin Urol. 2021 Jul 1;62(4):361–377. 10.4111/icu.20200602 PMC8246016 34085791

[15] Yang LS , Shan BL , Shan LL , Chin P , Murray S , Ahmadi N , et al. A systematic review and meta‐analysis of quality of life outcomes after radical cystectomy for bladder cancer. Surg Oncol. 2016 Sep;25(3):281–297. 27566035 10.1016/j.suronc.2016.05.027

[16] Rammant E , Fonteyne V , Van Goethem V , Verhaeghe S , Raes A , Van Hemelrijck M , et al. Supportive roles of the health care team throughout the illness trajectory of bladder cancer patients undergoing radical cystectomy: a qualitative study exploring the Patients' perspectives. Semin Oncol Nurs. 2021;37(6):151226. 34758914 10.1016/j.soncn.2021.151226

[17] Wan JCM . Survival outcomes of early versus deferred cystectomy for high‐grade non‐muscle‐invasive bladder cancer: a systematic review. Curr Urol. 2020;14(2):66–73. 32774230 10.1159/000499257 PMC7390979

[18] Li R , Sundi D , Zhang J , Kim Y , Sylvester RJ , Spiess PE , et al. Systematic review of the therapeutic efficacy of bladder‐preserving treatments for non–muscle‐invasive bladder cancer following intravesical bacillus Calmette‐Guérin. Eur Urol. 2020;78(3):387–399. 32143924 10.1016/j.eururo.2020.02.012 PMC7771323

[19] Murakami Y , Matsumoto K , Miyake M , Amano N , Shimura S , Nishimura N , et al. Real‐world treatment patterns and oncological outcomes in early relapse and refractory disease after bacillus Calmette‐Guérin failure in non‐muscle‐invasive bladder cancer. Int J Urol Off J Jpn Urol Assoc. 2022;29:1195–1203. 10.1111/iju.14976 35858755

[20] Miyake M , Kikuchi E , Shinozaki K , Piao Y , Hayashi N , Koto R , et al. Real‐world treatment patterns and clinical outcomes of Japanese patients with non‐muscle invasive bladder cancer receiving intravesical bacillus Calmette–Guérin treatment. Int J Urol. 2022;29(10):1120–1129. 35598101 10.1111/iju.14933 PMC9790662

[21] Packiam VT , Werntz RP , Steinberg GD . Current clinical trials in non‐muscle‐invasive bladder cancer: heightened need in an era of chronic BCG shortage. Curr Urol Rep. 2019;20(12):84. 31781942 10.1007/s11934-019-0952-y

[22] Hendricksen K , Aziz A , Bes P , Chun FKH , Dobruch J , Kluth LA , et al. Discrepancy between European Association of Urology guidelines and daily practice in the Management of non‐muscle‐invasive Bladder Cancer: results of a European survey. Eur Urol Focus. 2019;5(4):681–688. 29074050 10.1016/j.euf.2017.09.002

