# Supplementary figures and images for: Controversies in terminology associated with management of BCG‐unresponsive NMIBC in Asia‐Pacific
Source: Int J Urol. 2023 Oct 5;31(1):32–8. doi: 10.1111/iju.15298 (PMC11524088; doi:10.1111/iju.15298)

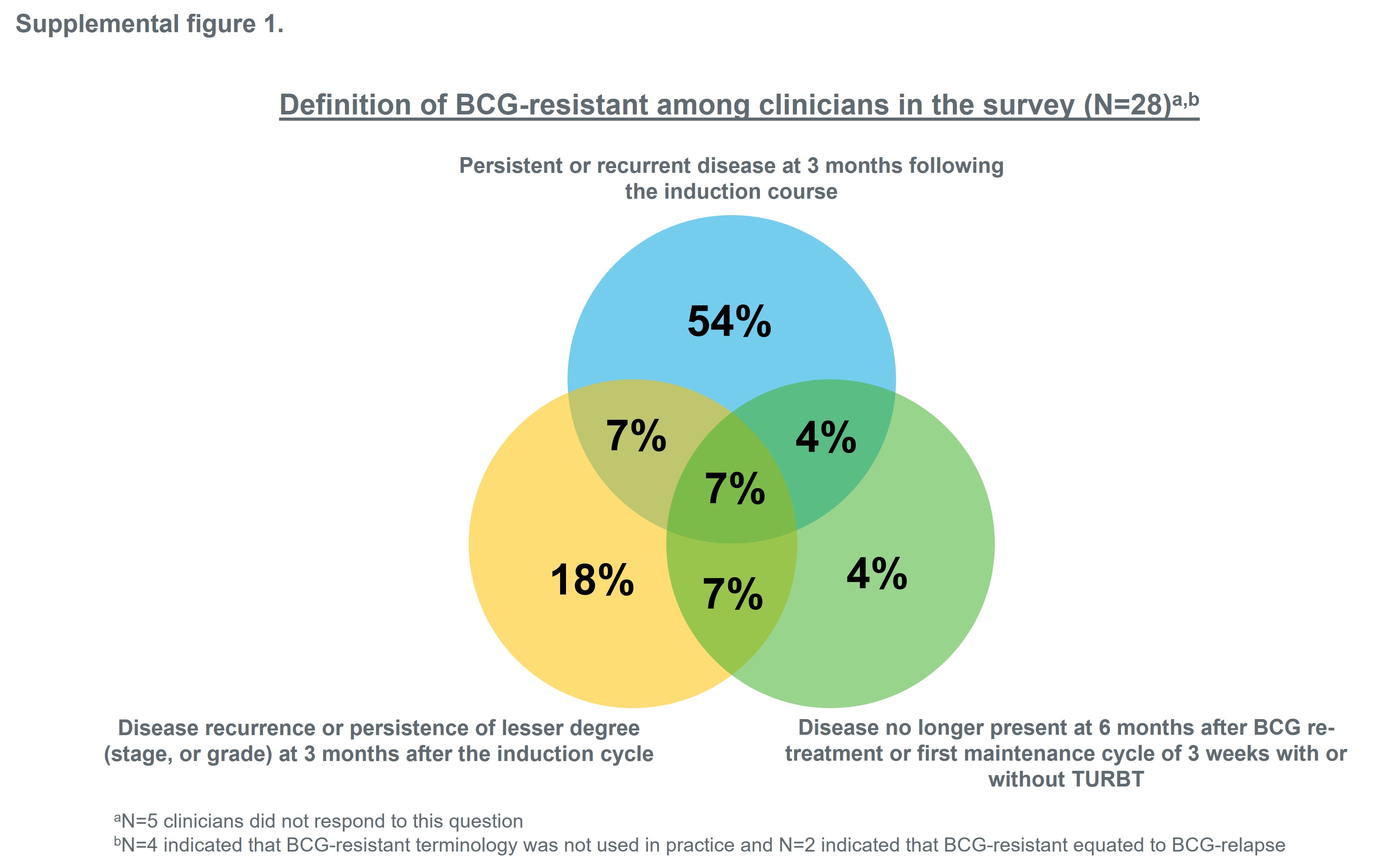

Supplement: Supplementary file 1 — Figure S1. [file IJU-31-32-s003.jpg]

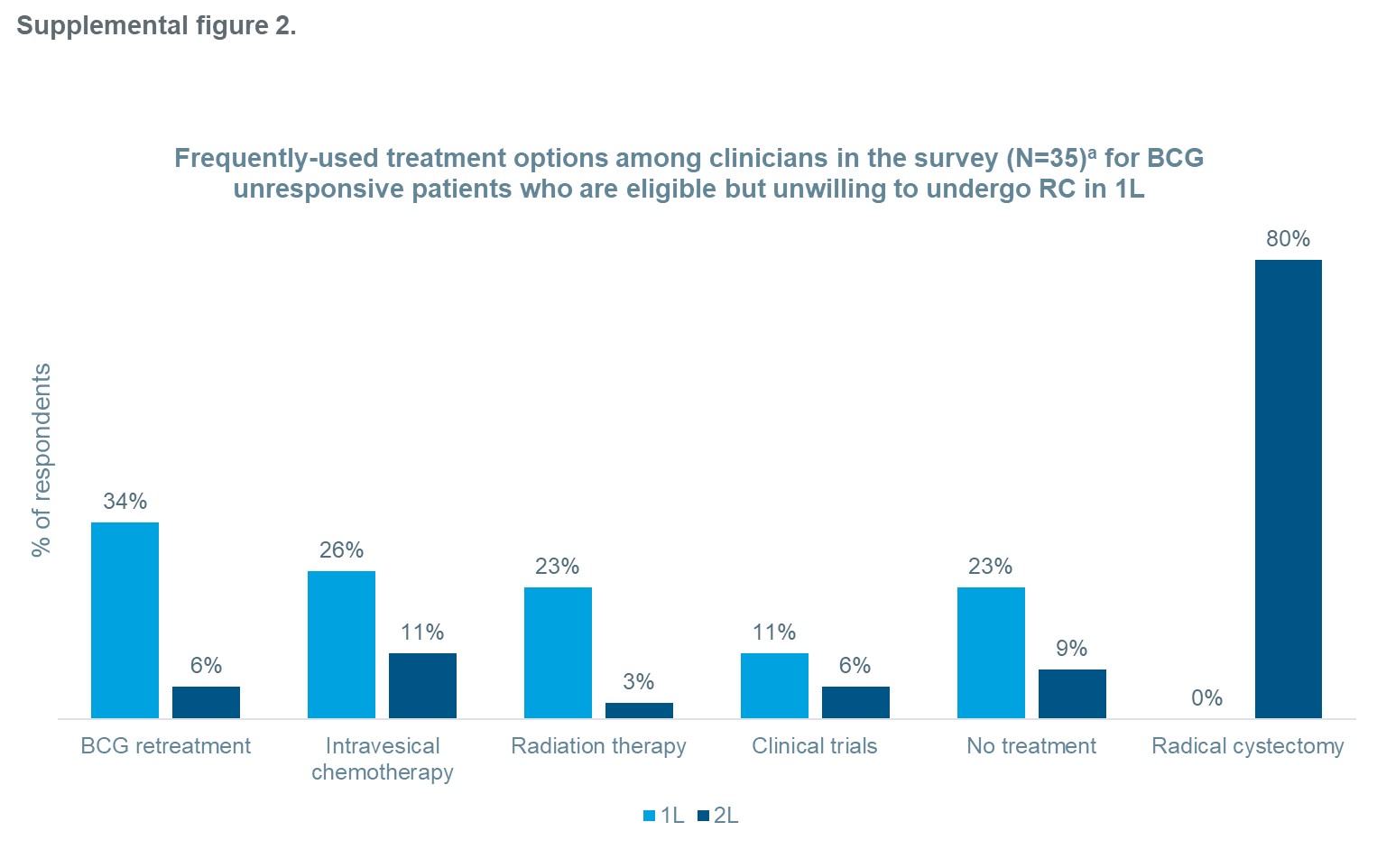

Supplement: Supplementary file 2 — Figure S2. [file IJU-31-32-s005.jpg]
