# Supplementary material for: Controversies in terminology associated with management of BCG‐unresponsive NMIBC in Asia‐Pacific
Source: Int J Urol. 2023 Oct 5;31(1):32–8. doi: 10.1111/iju.15298 (PMC11524088; doi:10.1111/iju.15298)
Supplement: Supplementary file 3 — Table S1. [file IJU-31-32-s001.docx]

**Supplemental Table 1**. Use of radical cystectomy and bladder-sparing treatment for BCG-unresponsive NMIBC patients who are willing and eligible to undergo RC among clinicians in the survey (N=39)

|  | Respondents (N) | Median (IQR) of patients who undergo RC | Median (IQR) of patients who receive bladder-sparing treatment |
| --- | --- | --- | --- |
| **Overall** | 39 | 50 (50-80) | 50 (20-50) |
| **Australia** | 8 | 55 (50-75) | 45 (25-50) |
| **Hong Kong** | 3 | 80 (80-85) | 20 (15-20) |
| **Japan** | 9 | 70 (50-80) | 30 (20-50) |
| **Singapore** | 5 | 50 (50-50) | 50 (50-50) |
| **Korea** | 8 | 50 (35-80) | 50 (20-65) |
| **Taiwan** | 6 | 40 (32.5-47.5) | 60 (52.5-67.5) |

IQR, interquartile range; RC, radical cystectomy
