# Supplementary material for: Controversies in terminology associated with management of BCG‐unresponsive NMIBC in Asia‐Pacific
Source: Int J Urol. 2023 Oct 5;31(1):32–8. doi: 10.1111/iju.15298 (PMC11524088; doi:10.1111/iju.15298)
Supplement: Supplementary file 4 — Data S1. [file IJU-31-32-s006.docx]

**Supplement 1: Definitions of BCG-treatment related terminology**

**EAU guidelines on NMIBC 2023**

**BCG-refractory tumor**

1. If T1 HG/G3 tumour is present at 3 months

2. If Ta HG/G3 tumour is present after 3 months and/or at 6 months, after either re-induction or first course of maintenance

3. If CIS (without concomitant papillary tumour) is present at 3 months and persists at 6 months after either re-induction or first course of maintenance. If patients with CIS present at 3 months, an additional BCG course can achieve a complete response in > 50% of cases

4. If HG tumour appears during BCG maintenance therapy. Patients with LG recurrence during or after BCG treatment are *not considered* to be a BCG failure.

**BCG-relapsing tumor**

Recurrence of HG/G3 (WHO 1973/2004) tumour after completion of BCG maintenance, despite an initial response

**BCG intolerance**

Severe side effects that prevent further BCG instillation before completing treatment

**BCG-resistant**

This terminology is not defined in the EAU guidelines. Several definitions have been proposed.

1. Persistent or recurrent disease at three months following the induction course *(Herr HW, Dalbagni G: Defining bacillus Calmette-Guerin refractory superficial bladder tumors. J. Urol. 2003; 169: 1706 –1708.)*
2. Disease no longer present at 6 months from BCG re-treatment with or without TURBT *(Kamel MH, Bailey SL, Moore JT, et al: Definition of BCG Failure in Non-Muscle Invasive Bladder Cancer in Major Urological Guidelines. UroToday Int J. 2011; 4: art82.)*
3. Disease recurrence or persistence of lesser degree, stage, or grade at 6 months from BCG re-treatment with or without TURBT *(Kamel MH, Bailey SL, Moore JT, et al: Definition of BCG Failure in Non-Muscle Invasive Bladder Cancer in Major Urological Guidelines. UroToday Int J. 2011; 4: art82.)*

**Definitions of BCG-unresponsive in various guidelines**

| EAU guidelines on NMIBC 2023 | BCG-unresponsive tumours include all BCG refractory tumours and those who develop T1/Ta HG recurrence within 6 months of completion of adequate BCG exposure or develop CIS within 12 months of completion of adequate BCG exposure.  Adequate BCG is defined as the completion of at least 5 of 6 doses of an initial induction course plus at least 2 of 6 doses of a second induction course, or 2 out of 3 doses of maintenance therapy. |
| --- | --- |
| JUA Clinical practice guidelines for bladder cancer 2019 | Generic term for BCG refractory and BCG-early relapsing (relapse within 12 months from last dose of BCG) |
| FDA guidance on BCG-unresponsive NMIBC 2018 | BCG-unresponsive disease is defined as being at least one of the following:   - Persistent or recurrent CIS alone or with recurrent Ta/T1 (noninvasive papillary disease/tumor invades the subepithelial connective tissue) disease within 12 months of completion of adequate BCG therapy - Recurrent high-grade Ta/T1 disease within 6 months of completion of adequate BCG therapy - T1 high-grade disease at the first evaluation following an induction BCG course |
| BCG-unresponsive NMIBC: recommendations from International Bladder Cancer Group 2017 | To be considered BCG-unresponsive, a patient must have received ≥1 induction course (6 weeks) and one maintenance course (3 weeks) and have either   - Refractory tumor (no disease-free interval) **or** - Recurrence of high-grade tumor within 6 months of their last BCG exposure |
