# Supplementary material for: Controversies in terminology associated with management of BCG‐unresponsive NMIBC in Asia‐Pacific
Source: Int J Urol. 2023 Oct 5;31(1):32–8. doi: 10.1111/iju.15298 (PMC11524088; doi:10.1111/iju.15298)
Supplement: Supplementary file 5 — Data S2. [file IJU-31-32-s002.docx]

**Supplement 2: Reference survey questions**

*Survey questions for reference*

1. Please select the characteristics that would be defined as BCG-resistant, if this terminology is used in your practice. Please select all relevant options.

| **BCG-resistant** | |
| --- | --- |
| This terminology is not used in my practice | 1 |
| Persistent or recurrent disease at 3 months following the induction course | 2 |
| Disease recurrence or persistence of lesser degree (stage, or grade) at 3 months after the induction cycle | 3 |
| Disease no longer present at 6 months after BCG re-treatment or first maintenance cycle of 3 weeks with or without TUR | 4 |
| Others, please specify | 5 |

1. Please indicate the characteristics that are considered BCG-unresponsive in your practice. Please select all relevant options.

| BCG-intolerant | 1 |
| --- | --- |
| BCG-refractory | 2 |
| BCG-relapsing | 3 |
| BCG-resistant | 4 |
| Others, please specify | 5 |

1. Please indicate the characteristics that are considered as BCG-failure in your practice. Please select all relevant options.

| This terminology is not used in my practice | 1 |
| --- | --- |
| BCG-intolerant | 2 |
| BCG-refractory | 3 |
| BCG-relapsing | 4 |
| BCG-resistant | 5 |
| Whenever MIBC is detected during follow-up | 6 |
| Others, please specify | 7 |


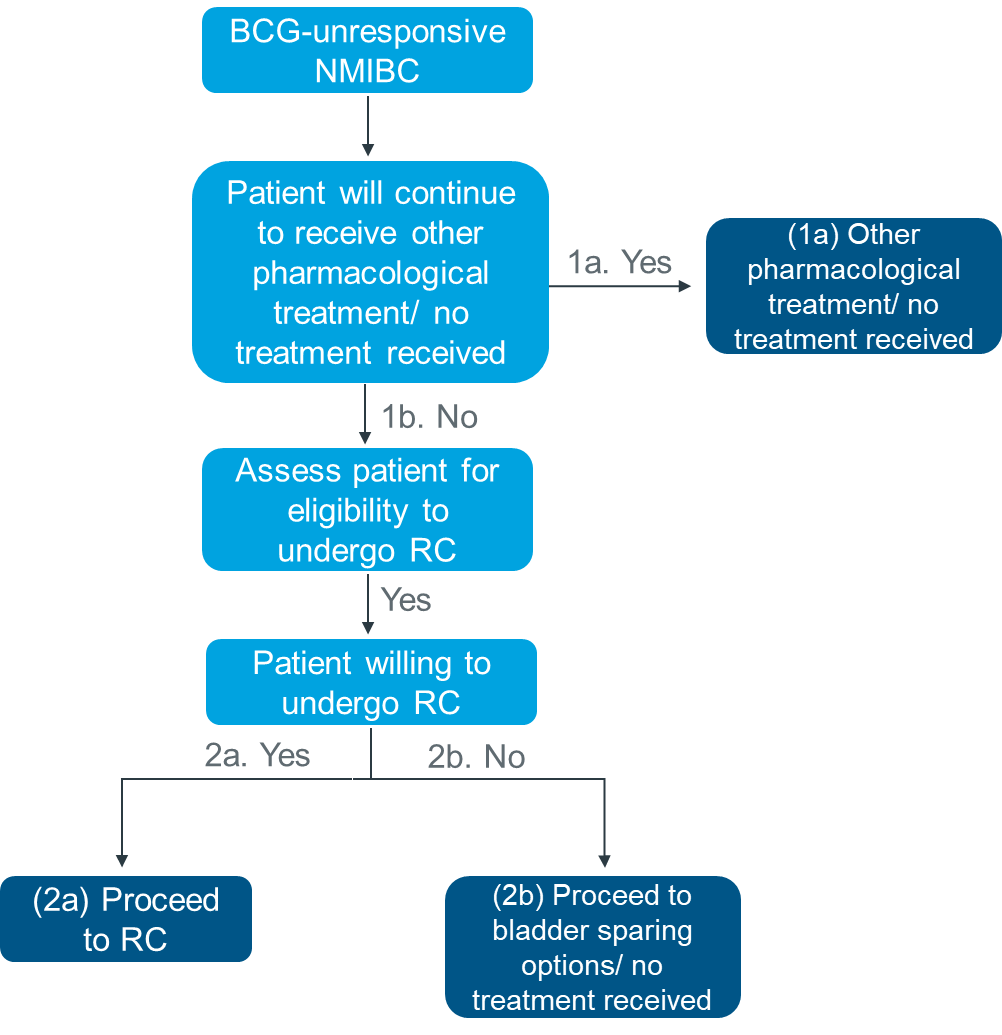


1. **Path 1**: Among BCG-unresponsive patients, what proportion of patients are (1a) selected and (1b) not selected to **continue to receive other pharmacological treatment/ no other treatment?**

| **Path** | **Option a** | **Option b** | **Total** |
| --- | --- | --- | --- |
| 1. |  |  | 100% |

**Path 2:** Among BCG-unresponsive patients who are **eligible for RC**, what proportion of patients are (2a) willing and (2b) unwilling to undergo RC?

| **Path** | **Option a** | **Option b** | **Total** |
| --- | --- | --- | --- |
| 2. |  |  | 100% |

Based on your definitions of BCG-unresponsive in question 15, please complete the table below for the following groups of **BCG-unresponsive patients** **who are eligible but unwilling for undergo RC**

Please indicate the corresponding proportion (%) of patients that receive the following management options (i) immediately post-BCG unresponsive (1L) and for (ii) subsequent treatment (2L and beyond):

1. Undergo radical cystectomy
2. Do not receive any further treatment
3. Respond to treatment used immediately post-BCG unresponsive or BCG-resistant (1L) and do not need subsequent treatment (2L and beyond)
4. Receive the following treatment options, from options *a* to *p*, as (i) immediate treatment post-BCG unresponsive (1L) and for (ii) subsequent treatment (2L and beyond)

*Note: All responses for each column should total to 100%*

| **Patients experiencing the following outcomes or receiving the following treatment** | **19. Pathway 2b: Are eligible but unwilling to undergo RC** | |
| --- | --- | --- |
|  | (i) % receiving this treatment **immediately post-BCG unresponsive (1L)** | (ii) % receiving this as **subsequent treatment after the 1L post-BCG unresponsive (2L and beyond)** |
| ***A. Undergo Radical cystectomy*** | **-** |  |
| ***B. No treatment*** |  |  |
| ***C. Patients respond to treatment used immediately post BCG-unresponsive/ resistant and do not require subsequent lines of treatment (2L and beyond)*** | **-** |  |
| ***D. Treatment options*** |  |  |
| ***SYSTEMIC*** |  |  |
| a. Clinical trials, please specify |  |  |
| ***b. RADIATION THERAPY*** |  |  |
| ***INTRAVESICAL*** |  |  |
| 1. ***BCG re-treatment*** |  |  |
| ***Cytotoxic*** |  |  |
| 1. Mitomycin |  |  |
| 1. Gemcitabine |  |  |
| 1. Doxorubicin |  |  |
| 1. Epirubicin |  |  |
| 1. Valrubicin |  |  |
| 1. Docetaxel |  |  |
| 1. Sequential gemcitabine/ docetaxel |  |  |
| 1. Sequential gemcitabine/ mitomycin |  |  |
| 1. Hyperthermic intravesical chemotherapy, please specify chemotherapy option |  |  |
| 1. Electromotive drug administration, please specify chemotherapy option |  |  |
| ***Immunogenic*** |  |  |
| 1. Instiladrin |  |  |
| 1. Bropirimine |  |  |
| 1. MCNA: *Mycobacterium phlei* cell wall-nucleic acid complex |  |  |
| 1. Anti-PD1/ PDL1 |  |  |
| ***OTHERS*** |  |  |
| 1. Others, please specify |  |  |
|  | 100% | 100% |
