# Supplementary material for: Controversies in terminology associated with management of BCG‐unresponsive NMIBC in Asia‐Pacific
Source: Int J Urol. 2023 Oct 5;31(1):32–8. doi: 10.1111/iju.15298 (PMC11524088; doi:10.1111/iju.15298)
Supplement: Supplementary file 6 — Data S3. [file IJU-31-32-s004.docx]

**Supplement 3: Reference in-depth interview questions**

*In-depth interview questions for reference*

1. Are you aware of the following terms?

If yes, is the term applied in (i) your usual clinical practice or (ii) as part of in your involvement in clinical trials? How would you define the terms in 1a to 1f?

To probe if not aware or used: Why is this term not used in usual clinical practice?

- 1. BCG-refractory
  2. Early BCG-relapse
  3. Late BCG-relapse
  4. BCG-unresponsive
  5. BCG-failure
  6. BCG-resistant

For the purposes of the flowchart below, **adequate BCG** is defined as **at least five of six doses of an initial induction course plus at least two of three doses of maintenance therapy***.*


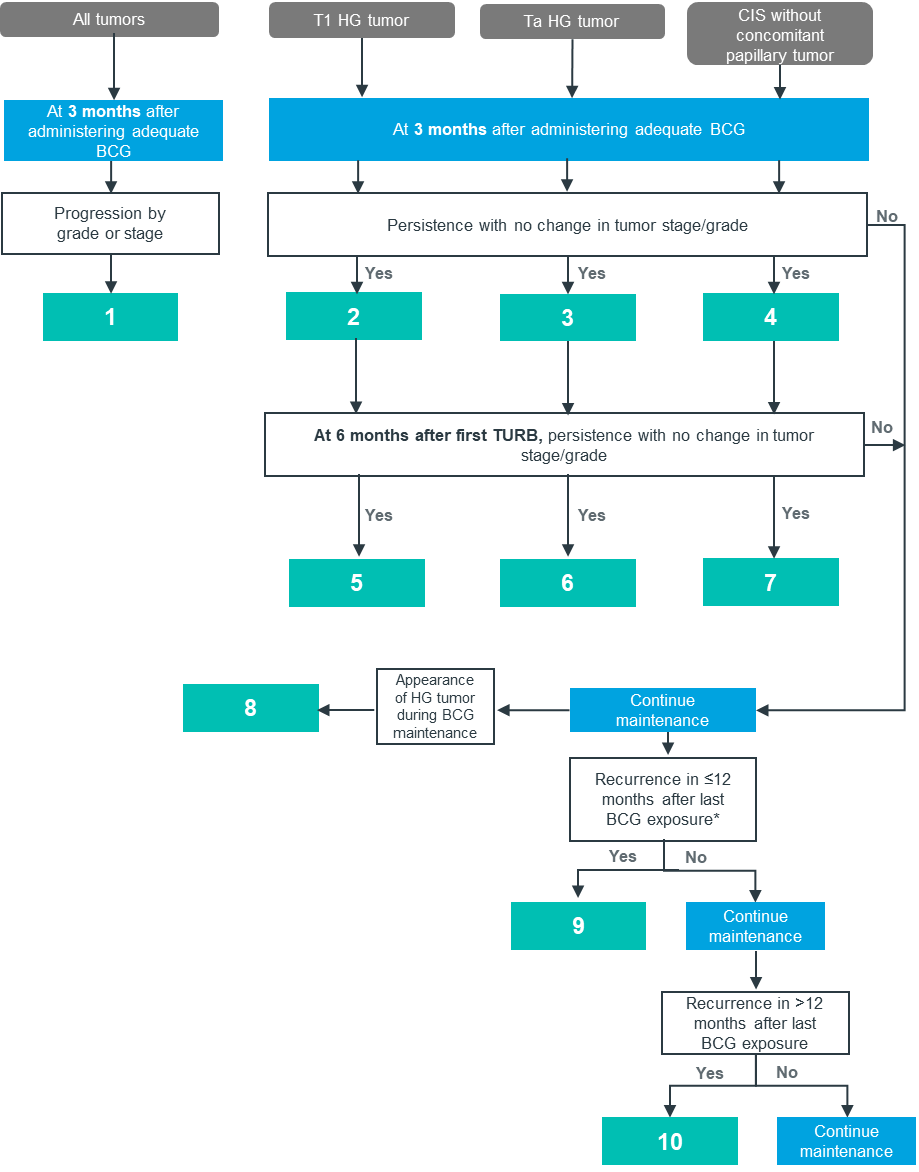


1. How would you manage the following characteristics numbered 1 to 10?
2. With reference to this diagram, please list all numbers you would classify as
   1. BCG-refractory
   2. Early BCG-relapse. Probe: In what duration after receiving adequate BCG is relapse considered **early** in your practice?
   3. Late BCG-relapse. Probe: In what duration after receiving adequate BCG is relapse considered **late** in your practice?
   4. BCG-unresponsive
   5. BCG-failure
   6. BCG-resistant [Skip this question if respondent does not use the term BCG-resistant]
3. The results from Round 1 of our survey showed that BCG-retreatment was the most frequently used option among BCG-unresponsive patients who continue to receive bladder-sparing treatment. Could you comment on why this might be so even when patients are deemed BCG-unresponsive?
